# Epidemiologic characteristics and influencing factors of cluster infection of COVID-19 in Jiangsu Province

**DOI:** 10.1017/S0950268821000327

**Published:** 2021-02-10

**Authors:** Jing Ai, Naiyang Shi, Yingying Shi, Ke Xu, Qigang Dai, Wendong Liu, Liling Chen, Junjun Wang, Qiang Gao, Hong Ji, Ying Wu, Haodi Huang, Ziping Zhao, Hui Jin, Changjun Bao

**Affiliations:** 1 Department of Acute Infectious Diseases Control and Prevention, Jiangsu Provincial Centre for Disease Control and Prevention, Nanjing, China; 2 Department of Epidemiology and Health Statistics, School of Public Health, Southeast University, Nanjing, China; 3 Key Laboratory of Environmental Medicine Engineering, Ministry of Education, Nanjing, China; 4 Suzhou Centre for Disease Control and Prevention, Suzhou, China; 5 Nanjing Centre for Disease Control and Prevention, Nanjing, China; 6 Huaian Centre for Disease Control and Prevention, Huaian, China; 7 School of Public Health, Nanjing Medical University, Nanjing, China; 8 NHC Key Laboratory of Enteric Pathogenic Microbiology, Nanjing, China

**Keywords:** Cluster infection, COVID-19, epidemiology, secondary infection

## Abstract

To understand the characteristics and influencing factors related to cluster infections in Jiangsu Province, China, we investigated case reports to explore transmission dynamics and influencing factors of scales of cluster infection. The effectiveness of interventions was assessed by changes in the time-dependent reproductive number (*R_t_*). From 25th January to 29th February, Jiangsu Province reported a total of 134 clusters involving 617 cases. Household clusters accounted for 79.85% of the total. The time interval from onset to report of index cases was 8 days, which was longer than that of secondary cases (4 days) (*χ*^2^ = 22.763, *P* < 0.001) and had a relationship with the number of secondary cases (the correlation coefficient (*r*) = 0.193, *P* = 0.040). The average interval from onset to report was different between family cluster cases (4 days) and community cluster cases (7 days) (*χ*^2^ = 28.072, *P* < 0.001). The average time interval from onset to isolation of patients with secondary infection (5 days) was longer than that of patients without secondary infection (3 days) (*F* = 9.761, *P* = 0.002). Asymptomatic patients and non-familial clusters had impacts on the size of the clusters. The average reduction in the *R_t_* value in family clusters (26.00%, 0.26 ± 0.22) was lower than that in other clusters (37.00%, 0.37 ± 0.26) (*F* = 4.400, *P* = 0.039). Early detection of asymptomatic patients and early reports of non-family clusters can effectively weaken cluster infections.

## Introduction

Novel coronaviruses, including severe acute respiratory syndrome coronavirus (SARS-CoV) in 2002 and middle east respiratory syndrome coronavirus (MERS-CoV) in 2012, have led to large-scale epidemics [1, 2]. A novel coronavirus pneumonia (COVID-19) appeared in 2019 and spread rapidly across China [3]. Compared with SARS-CoV and MERS-CoV, SARS-CoV-2 has stronger transmissibility [2–5], which facilitates cluster infection.

To date, many countries and territories have reported cluster infections of COVID-19. A large number of authors has elaborated on the transmission chain and epidemiological characteristics of one cluster infection [5–11]. Additionally, observational studies have included regional cluster infections and analysed the epidemiological characteristics of the involved cases [12–14]. The literature provides valuable evidence for COVID-19, but the influencing factors related to cluster infections have rarely been discussed. To understand the characteristics and influencing factors related to cluster infections, our study summarised the epidemiological characteristics of cluster infections in Jiangsu Province and deeply investigated case reports to explore transmission dynamics and influencing factors of scales of cluster infection to provide a better basis for the formulation of prevention and control measures.

## Methods

### Data source

The cluster infection data were collected from the ‘Public Health Emergency Information Management System’ designed by China's Disease Prevention and Control Centre. The index cases were based on individuals went to a hospital, while the close contacts of index cases, some of which then developed as secondary cases, were identified during the investigation of index cases. If the close contacts tested positive, they would be isolated and receive treatment in hospital. We reviewed epidemiological investigation reports and extracted key information for analysis. We summarised transmission chain according to the date of exposure, illness onset and isolation, the place of exposure, etc. The sequence was the same as the report we published before [15].

### Subjects and definitions

We referred to the COVID-19 Prevention and Control Program (Third Edition) and defined cluster infection as infections involving two or more confirmed cases or asymptomatic-infected cases in small units (family, construction site, affiliation, etc.) within 14 days and the possibility of interpersonal transmission due to close contact or common source exposure [16]. The subjects included in this study were as follows.

Index cases: Cases transmitting the virus to others in a cluster infection.

Secondary cases: Cases infected by index cases.

Common source exposure: Cases with the same travel or residency history in Hubei Province or other infected areas and that did not confirm the transmission path; these cases were excluded from the index cases and secondary cases.

Intergeneration: The judgement of intergenerational cases (e.g., first, second or third generation) was based on China's Guidelines for Epidemiological Investigation of Novel Coronavirus Pneumonia Cluster Infection.

Cluster scale: We defined different cluster scales according to the number of cases and specifically categorised them into small-scale clusters (<10 cases) and large-scale clusters (≥10 cases).

Onset time: Time of symptom appearance.

### Pathogen detection

Reverse transcription-polymerase chain reaction (RT-PCR) and/or high-throughput sequencing (next-generation sequencing) were applied for SARS-CoV-2 nucleic acid detection in nasopharyngeal swabs, sputum and other lower respiratory tract secretions and blood and faecal specimens. Positive laboratory cases required at least one of the following two conditions: (1) two targets (ORF1ab, N) in the same specimen had both positive RT-PCR results; if only a single-target test result was positive, resampling and retesting were required and if the retest result was the same as the result of the first test, the specimen could be defined as positive; and (2) both specimens showed a positive single-target RT-PCR result, or the test result of a single-target positive in two sampling tests of the same type of specimen could be determined as positive.

### Statistical analysis

SPSS 23.0 software was used for data statistics and analysis. For categorical variables, the chi-square (*χ*^2^) test was conducted for comparisons between rates. For continuous variables, *F*-tests and non-parametric tests were conducted for comparisons between groups. R4.0.1 software was used for the univariate and multivariate analysis of the factors affecting the scale of cluster infection, which were carried out by random-effects logistic regression. Random-effects logistic regression model was applied to explore the association between the scale of the cluster epidemic and potential factors such as age and sex. The data analysis strategy is as follows: each observation represented an individual in a cluster. The dependent variable was the size of the cluster (small/large was coded as 0/1) and the cluster was treated as the random-effect variable. The effectiveness of interventions was assessed by changes in the time-dependent reproductive number (*R_t_*) [17], and the formula is as follows:
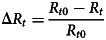
*R_t_* refers to the *R_t_* value of each cluster at the end, and *R_t_*_0_ refers to the *R_t_* value of each cluster when the first case was observed. The calculation of the *R_t_* value was carried out by the R package ‘EpiEstim’ [18]. We drew a curve of the *R_t_* value in each city and brought family clusters and the other clusters into the corresponding curves, estimating the prevention and control strength (Δ*R*_*t*_). ArcGIS10.0 was applied for map drawing.

## Results

From 25th January to 29th February, Jiangsu Province reported a total of 134 cluster infections involving 617 cases. Small-scale clusters (2–4 cases) accounted for 74.63% of the total cluster infections and 42.46% of the total cases.

### Time distribution

**Fig. 1.**
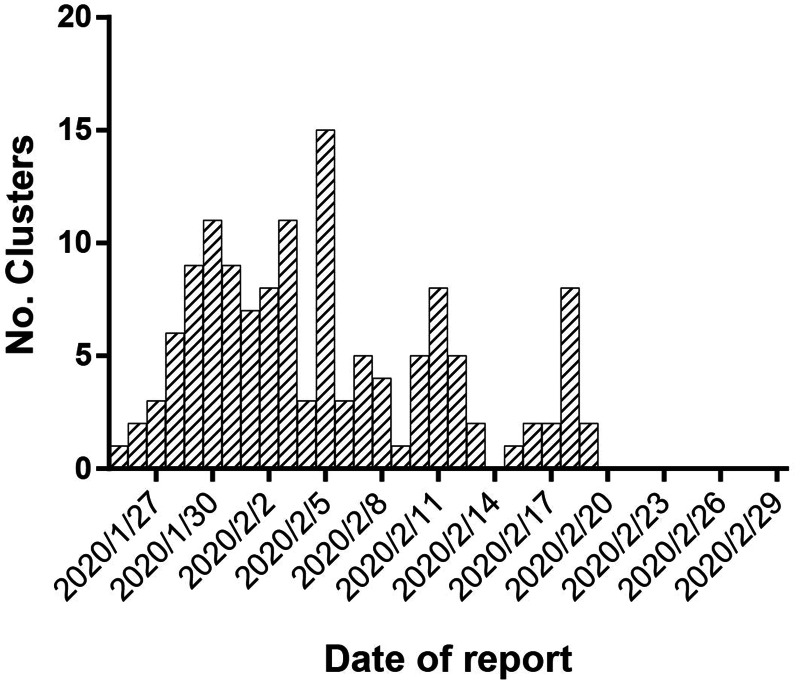
Time distribution of cluster infection in Jiangsu Province.

On 25th January, the first cluster infection was reported. The first case had a history of Wuhan residence and caused a family cluster infection after returning. In the following 11 days, the number of outbreaks increased significantly and reached the peak of daily reports on 5th February (15, 11.19%). Since 5th February, Jiangsu carried out concentrated observations on close contacts, and the number of epidemic reports showed a significant fluctuating downward trend. The last two infections were reported on 19th February (Fig. 1).

### Regional distribution

**Fig. 2.**
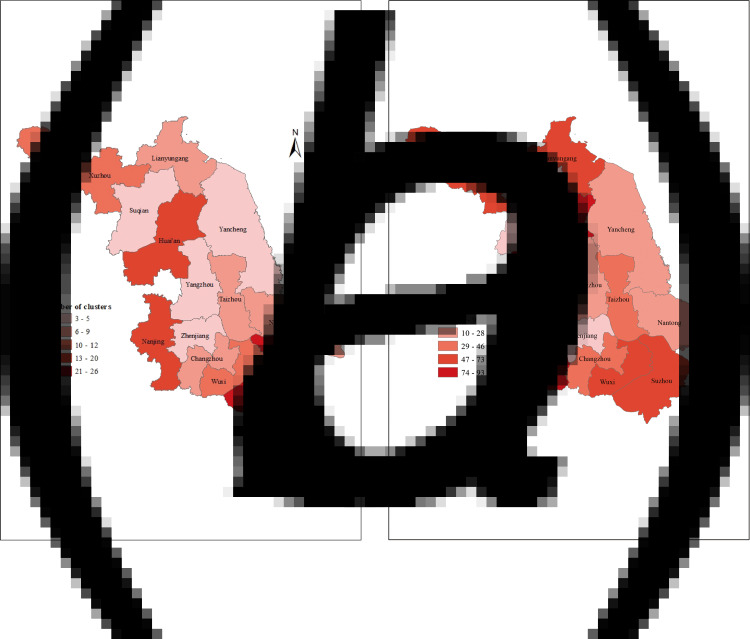
GIS map of the city distribution of COVID-19 cluster infection in Jiangsu Province (a). The number of COVID-19 cluster infections (b). The number of COVID-19 cluster cases.

Among 13 cities, Suzhou (19.41%, 26/134), Nanjing (14.93%, 20/134) and Huai'an (13.43%, 18/134) accounted for nearly half of the cluster infections, while Huai'an (15.07%, 93/617), Nanjing (14.26%, 88/617) and Xuzhou (11.83%, 73/617) showed a high incidence given the total number of infected cases. For the scale of clusters, Lianyungang (9.71 cases/infection), Yancheng (6.75 cases/infection) and Xuzhou (6.63 cases/infection) ranked at the top. The details are shown in Figure 2.

### Population distribution

A total of 607 cases were involved in the cluster infection, with a sex ratio of 0.93 (male:female = 292:315) and an average age of 44.11 years. Further analysis of gender indicated a significant difference between index cases and secondary cases (*χ*^2^ = 13.936, *P* < 0.001); most of the index cases involved males (63.64%), while the majority of secondary cases involved females (56.54%). There was no significant difference in age between index cases and secondary cases, with an average age of 44.35 and 45.02 years old, respectively.

### Site distribution

The vast majority of cluster outbreaks occurred within families (eating and living together, visiting relatives, etc.), accounting for 79.85% (107/134) of the total. Seven (5.22%, 7/134) clusters were observed in the community (playing cards with neighbourhood friends, leisure in public bathrooms, neighbourhood or friend communication, etc.). Three (2.24%, 3/134) clusters were observed in the context of work affiliations (meeting, co-office, etc.). Noticeably, there were 17 (12.69%, 17/134) clusters of cross-site transmission, 7 (41.18%) clusters of transmission from family to friends, 6 clusters of transmission from work affiliation to family (35.29%), 2 clusters of transmission from family to work affiliation (11.76%) and 2 clusters of other types (11.76%). Cluster infections with more than 20 cases mainly occurred in communities or work affiliations.

### Travel and residential history

The distance between Wuhan (capital city of Hubei Province) and Nanjing (capital city of Jiangsu Province) is about 550 km. It takes about 3 h by train, or 7 h when self-driving. The Spring Festival transportation increased the round-trip passenger flow between the two provinces. Among the reported cluster infections, 59 (44.03%, 59/134) local transmission clusters were the result of travelling or residing in other provinces or abroad, among which 44 (74.58%) clusters' index cases had a travel or residential history in Hubei Province, 10 (16.95%) clusters' index cases had a travel or residential history in other provinces and 5 (8.47%) clusters' index cases had a travel or residential history abroad. The cases involved in 36 (26.87%, 36/134) clusters all had a travel or residential history in Hubei Province, other provinces or abroad but did not cause local transmission.

The whole cluster epidemic could be divided into two stages. Of the 83 clusters reported from 25th January to 5th February, 53 (63.86%) were caused by imported cases in Hubei Province. In the later stage, however, the proportion from 6th February to 19th February was 21.56%, which decreased by 66.24% from the previous stage, and the infections caused by local transmission began to dominate.

Further statistical analysis demonstrated that the travel or residential histories of index cases had no effect on cluster size (*t* = −0.636, *P* = 0.526) or the clinical severity of secondary cases (*χ*^2^ = 0.190, *P* = 1.000).

### Onset time interval of different intergenerational cases

The average onset time interval between the first generation and the second generation was 6.22 ± 5.67 (95% confidence interval (CI) 0.55–11.89) days, and that between the second and third generation was 5.60 ± 6.02 (95% CI −0.42 to 11.62) days. The statistical analysis suggested no significant difference between the generations.

### Effects of early detection, early reporting and early isolation of cases on cluster infections

The average time interval from onset to report of index cases was 8 days, which was longer than that of secondary cases (4 days) (*χ*^2^ = 22.763, *P* < 0.001, Table 1). The correlation coefficient between the time interval from onset to report of an index case and the number of secondary cases was 0.193 (*P* = 0.040). The results showed a significant difference in the average interval between family cluster cases and community cluster cases, which was 4 and 7 days, respectively (*χ*^2^ = 28.072, *P* < 0.001) (Table 1).

**Table 1.** Time intervals between onset and report and between onset and isolation (days)

Time interval (start-end)	Groups	*n*	*N* (*Q*_25_–*Q*_75_)	*Z*	*P*
Onset-report	Index cases	110	8.00 (4.00–10.00)	22.763	<0.001
	Secondary cases	382	4.00 (2.00–8.00)		
Onset-report	Family cluster cases	374	4.00 (2.00–8.00)	28.072	<0.001
	Community cluster cases	66	7.00 (5.00–11.00)		
Onset-isolation	Patients without secondary cases	232	3.00 (0.00–6.00)	9.761	0.002
	Patients with secondary cases	110	5.00 (1.00–8.00)		

*M* ± s.d., mean ± standard deviation.

The average time interval from onset to isolation of patients with secondary cases was 5 days, which was longer than the 3-day interval for patients without secondary cases, and there was a significant difference between the two groups (*F* = 9.761, *P* = 0.002).

### Comparison of clinical severity between index cases and secondary cases

There was a significant difference in the clinical severity between the index cases and the secondary cases (*χ*^2^ = 9.677, *P* = 0.008). Among the index cases, 61.8% had common pneumonia, which was much higher than the proportion of mild and asymptomatic infections and severe pneumonia. Among the secondary cases, the proportion of common pneumonia (48.7%) was roughly the same as that of mild or asymptomatic infections (47.1%). The proportion of severe pneumonia in index cases (7.3%) was much higher than that in secondary cases (4.2%). The details are shown in Table 2.

**Table 2.** Clinical severity of index cases and secondary cases (*N* (%))

Groups	Clinical severity			*χ^2^*	*P*
	Severe pneumonia	Common pneumonia	Mild pneumonia or asymptomatic infections		
Index cases	8 (7.3)	68 (61.8)	34 (30.9)	9.677	0.008
Secondary cases	16 (4.2)	186 (48.7)	180 (47.1)		
Total	29 (4.8)	307 (50.6)	271 (44.6)		

### Risk factors for cluster infection scales

Excluding cases imported from other provinces, 103 clusters (492 cases) were selected from 134 outbreaks. The infections were divided into two scales of clusters: small-scale clusters (clusters of less than 10 cases; 299 cases in total, 60.77%) and large-scale cases (clusters of at least 10 cases; 193 cases in total, 39.23%).

Taking two groups of different scales with dependent variables (including sex, age, occupation, gathering site, time interval from case onset to treatment, time interval from onset to report, time interval from onset to isolation, case classification, the number of close contacts of index cases, the region and whether or not fever developed) as independent variables, the results of univariate analysis indicated that occupation, gathering site, the time interval from onset to treatment and case classification were statistically significant (Table 3). Further multivariate analysis incorporating factors whose *P* value <0.1 demonstrated that case classification and gathering site had impacts on the size of the clusters (Table 4).

**Table 3.** Univariate logistic analysis of risk factors for cluster infection scales (No. (%))

Factors	2–9	≥10	Statistics	*P*	OR	OR 95% CI
Sex			0.08	0.780		
Male	136 (46.9%)	97 (48.7%)				
Female	154 (53.1%)	102 (51.3%)	−0.28	0.780	0.929	0.553–1.559
Age			6.02	0.111		
1–18	23 (7.9%)	5 (2.5%)				
19–40	94 (32.4%)	72 (36.2%)	2.21	0.027	3.523	1.155–10.750
41–65	137 (47.2%)	100 (50.3%)	2.42	0.015	3.358	1.261–8.938
≥66	36 (12.4%)	22 (11.1%)	1.96	0.050	2.811	0.999–7.909
Occupation			3.45	0.063		
Medical workers	12 (4.1%)	28 (14.1%)	–			
Non-medical workers	278 (95.9%)	171 (85.9%)	−1.86	0.063	0.264	0.065–1.077
Gathering site			13.57	0.004		
Family	217 (74.18%)	41 (20.6%)	–			
Community	14 (4.8%)	52 (26.1%)	2.75	0.006	19.659	2.359–163.819
Working affiliation	14 (4.8%)	57 (28.6%)	2.88	0.004	21.549	2.662–174.430
Cross-sites	45 (15.5%)	49 (24.6%)	2.17	0.030	5.763	1.185–28.019
Onset-treatment	1.00 (0.00–4.00)	2.00 (0.00–5.00)	−2.17	0.030	0.992	0.986–0.999
Onset-report	5.00 (2.00–8.00)	6.00 (3.00–9.00)	1.22	0.223	1.038	0.978–1.101
Onset-isolation	1.00 (0.00–5.00)	3.00 (0.00–5.00)	0.49	0.625	1.016	0.952–1.085
Classification			3.25	0.071		
Confirmed cases	222 (76.6%)	128 (64.3%)	–			
Asymptomatic cases	68 (23.4%)	71 (35.7%)	1.80	0.071	1.81	0.95–3.45
Close contacts of index cases	22 (6–49)	38 (34–119)	1.60	0.109	1.015	0.997–1.034
Fever			1.09	0.296		
No	112 (38.6%)	90 (45.2%)				
Yes	178 (61.4%)	109 (54.8%)	−1.05	0.296	0.762	0.458–1.268

OR, odds ratio; CI, confidence interval.

**Table 4.** Multivariate logistic model analysis of risk factors for cluster infection scales

Factors	Statistics	*P*	OR	OR 95% CI
Occupation				
Medical workers	1			
Non-medical workers	−0.89	0.374	0.592	0.186–1.883
Gathering site				
Family	1			
Community	2.35	0.019	13.033	1.532–110.863
Working affiliation	2.80	0.005	21.449	2.516–182.985
Cross-sites	1.75	0.080	4.567	0.836–24.945
Onset-treatment	1.71	0.088	1.087	0.988–1.197
Classification				
Confirmed cases	1			
Asymptomatic cases	2.65	0.008	2.71	1.298–5.651

OR, odds ratio; CI, confidence interval.

Control groups: medical workers, family, confirmed cases.

### Evaluation of the effects of prevention and control measures in different gathering sites

The results showed that the average reduction of the *R_t_* value in family clusters (26.00%, 0.26 ± 0.22) was lower than that in other clusters (37%, 0.37 ± 0.26), and the difference was statistically significant (*F* = 4.400, *P* = 0.039).

## Discussion

The outbreak of COVID-19 is another major public health event that China has encountered since SARS in 2002. Given its higher transmissibility, it is easier to attribute cluster infections to COVID-19. Our study included 617 cases of COVID-19 cluster infections in Jiangsu Province and determined the epidemiological characteristics and influencing factors of the clusters.

The cluster infections in Jiangsu Province were mainly concentrated between late-January and mid-February, divided into two stages according to the transmission characteristics. The first stage, occurring from 25th January to 5th February, gave rise to clusters caused by the imported cases in Hubei Province, which was probably because of the Spring Festival travel rush. The 25th January was the Chinese Lunar New year, and the previous 1–2 weeks were the peak period for gatherings. Although Wuhan city had been closed since 23rd January, a large number of people from Hubei Province had left before the closure of the city. Infected individuals could infect others during the incubation period even if they were asymptomatic, leading to local transmission [19–22]. However, during the second stage from 6th to 19th February, with the substantial reduction in personnel mobility and the continuous strengthening of prevention and control measures, the cluster infections decreased by 38.55% and converted to local transmission [23, 24].

The regional characteristics were also meaningful. According to the city's local site, Jiangsu Province can usually be divided into three regions: southern Jiangsu, central Jiangsu and northern Jiangsu. In terms of the average cluster size, northern Jiangsu was more sensitive, which may be associated with the differences in lifestyle habits among different regions; those in northern Jiangsu had more frequent social contact and social activities, for example, residents in northern Jiangsu like to play cards and take bath in public bathing pools. The difference in prevention and control efforts among different regions may also be the reason.

Our study found that the cases mainly occurred among middle-aged males, which is a finding similar to that observed in a study involving 1052 COVID-19 cluster cases [25] but inconsistent with that of the study of the initial 425 cases and an analysis of the first 99 cases in Wuhan, where most cases were middle-aged and elderly males, rather than only middle-aged [26, 27]. The sex distribution showed that most index cases involved males, but the majority of secondary cases involved females. The result may be related to the possibility that males were more interested in social activities involving several people, which could lead to a higher chance of infection. It is worth noting that the sample size may also bias the conclusion. Given the relatively small size of these outbreaks that were primarily restricted to homes, we could not rule out the possibility of the influence of household composition. For example, if a household is made up of approximately 50% males and females, then a majority of index cases being males must lead to secondary cases being females.

Nearly 80% of cluster infections occurred within families through living or eating together, which is consistent with the report at the press conference of the Joint Prevention and Control Mechanism of the State Council on 11th February that family clusters accounted for 83% of nearly 1000 cluster outbreaks [28]. Previous studies reported that the attack rate was highest among populations living together, and family members were at the highest risk [29]. Close contacts were allowed to be quarantined at home before 5th February, which allowed for the possibility of family contact due to the limitation of home isolation conditions, even though information on the requirements for home isolation was provided. As a result, family cluster infections were difficult to avoid, indicating that the effect of home isolation was limited [30]. Since 5th February, however, family cluster infections had shown a downward trend under the circumstance that Jiangsu Province implemented single-room centralised isolation for close contacts, indicating that the effect of strict centralised single-room observation measures for close contacts was better.

The time interval from onset to report of secondary cases was shorter than that of index cases, which is consistent with the results of 1052 cases [25]. The results indicated that the time for the detection of cases was gradually shortened. The time interval from onset to report of the index cases was positively correlated with the number of secondary cases. In addition, the average time interval from onset to isolation of cases with secondary infection was longer than that of cases without secondary infection. The above results revealed that early detection, early reporting and early isolation of cases are of great significance in reducing secondary infection, and prevention and control measures can reduce the risk of retransmission.

The results of multivariate analysis showed that the gathering sites and classification of cases influenced the scale of the infection. Small-scale clusters were dominated by family (74.18%), while the proportion of community, unit and cross-site clusters increased significantly with the increase in the scale of the clusters. The assessment of prevention and control measures based on communication dynamics further demonstrated that interventions were more effective in public sites such as communities or units than in families, but it was still difficult to decrease the scale of infection. This result suggested that once the infection occurred in communities, units or other public sites, it could easily lead to large-scale cluster infections regardless of the strength of interventions. The time interval from onset to report was significantly higher in community clusters than in family clusters, suggesting that the detection of cases in public sites was more difficult and untimely, which increased the risk of transmission and the difficulty of prevention work [31, 32], which proved to be highly significant in strengthening the detection of cases or suspected cases and screening and isolating close contacts. Based on the results, public gatherings should not be encouraged, and crowded places should be avoided. Meanwhile, wearing masks and washing hands should be promoted to decrease the chances of infection. The analysis also found that with the increase in the proportion of asymptomatic infection, the scale of the cluster was on the rise, while the proportion of confirmed cases decreased accordingly, suggesting that asymptomatic infection should not be ignored [20, 31].

Although occupation and the time interval from onset to treatment were insignificant in multivariate analysis, they are still worthy of attention. In addition to being a group with a high risk of infection, medical workers themselves also pose risks of large-scale cluster infections [33]. Hospitals are crowded spaces where relationships with visitors are complex and should be focused on. Medical workers must focus on personal protection during diagnosis and nursing procedures to avoid infection by patients and further cross-infection. Disinfection and biosafety protection work are also important, as well as the optimisation of the layout of the hospital fever clinic and medical treatment process, aimed at preventing nosocomial infection as much as possible. The time interval from onset to treatment of cases in large-scale clusters was longer than that in small-scale clusters, indicating that early consultation and diagnosis could reduce the spread and scale of infection, which is also mentioned in a previous study using dataset from the USA, Korea and European countries [34].

There were some limitations in our study. First, the proportion of mild pneumonia and asymptomatic infection in secondary cases was higher than that in index cases, while the proportion of severe pneumonia was lower, which is similar to the results of a study in Shenzhen [30]. However, this conclusion requires further verification due to the small sample size. Additionally, in the analysis of the risk factors affecting the scale of infection, the number of close contacts was not significant, which may be related to differences in the intensity and assurance of the investigation in different places. Last but not least, COVID-19 is a complex of medical conditions, and not a cause. The ultimate causative agent is not a virus in isolation, but a virus in complex with particular social factors [35]. As COVID-19 is an ongoing pandemic, it has possibility that our conclusion might be reversed in future.

## Conclusions

Early detection, early reporting and early isolation can effectively weaken cluster infections. With the gradual resumption of work and education, the monitoring and registration of fever, cough, abdominal pain and diarrhoea, as well as the screening of suspected cases, should be prioritised to facilitate the early detection, reporting and treatment of cases to reduce secondary cases and slow down outbreaks, thus lowering the pressure placed on medical services and social operations due to COVID-19.

## Data Availability

The datasets generated and/or analysed during the current study are not publicly available due to privacy or ethical restrictions, but are available from the corresponding authors on reasonable request.

## References

[1] Drosten C (2003) Identification of a novel coronavirus in patients with severe acute respiratory syndrome. New England Journal of Medicine 348, 1967–1976. 10.1056/NEJMoa030747 12690091

[2] Zaki AM (2012) Isolation of a novel coronavirus from a man with pneumonia in Saudi Arabia. New England Journal of Medicine 367, 1814–1820. 10.1056/NEJMoa1211721 23075143

[3] Wang C (2020) A novel coronavirus outbreak of global health concern. Lancet 395, 470–473. 31986257 10.1016/S0140-6736(20)30185-9 PMC7135038

[4] Munster VJ (2020) A novel coronavirus emerging in China – key questions for impact assessment. New England Journal of Medicine 382, 692–694. 10.1056/NEJMp2000929 31978293

[5] Chan JF-W (2020) A familial cluster of pneumonia associated with the 2019 novel coronavirus indicating person-to-person transmission: a study of a family cluster. Lancet 395, 514–523. 31986261 10.1016/S0140-6736(20)30154-9 PMC7159286

[6] Huang R (2020) A family cluster of SARS-CoV-2 infection involving 11 patients in Nanjing, China. The Lancet Infectious Diseases 20, 534–535. 10.1016/S1473-3099(20)30147-X PMC7159019 32119823

[7] Luo C (2020) Possible transmission of severe acute respiratory syndrome coronavirus 2 (SARS-CoV-2) in a Public Bath Center in Huai'an, Jiangsu Province, China. JAMA Network Open 3, e204583. 32227177 10.1001/jamanetworkopen.2020.4583 PMC12543385

[8] Weishen W (2020) Investigation and analysis on characteristics of a cluster of COVID-19 associated with exposure in a department store in Tianjin. Chinese Journal of Epidemiology 41, 489–493. 32133830 10.3760/cma.j.cn112338-20200221-00139

[9] Böhmer MM (2020) Investigation of a COVID-19 outbreak in Germany resulting from a single travel-associated primary case: a case series. Lancet Infectious Disease 20, 920–928. 10.1016/S1473-3099(20)30314-5 PMC7228725 32422201

[10] Jang S, Han SH and Rhee J-Y (2020) Cluster of coronavirus disease associated with fitness dance classes, South Korea. Emerging Infectious Diseases 26, 1917–1920. 32412896 10.3201/eid2608.200633 PMC7392463

[11] Hodcroft EB (2020) Preliminary case report on the SARS-CoV-2 cluster in the UK, France, and Spain. Swiss Medical Weekly 150, 9–10. 10.4414/smw.2020.20212 32227799

[12] Cheongju (2020) Coronavirus disease-19: summary of 2370 contact investigations of the first 30 cases in the Republic of Korea. Osong Public Health Research Perspectives 11, 81–84. 32257773 10.24171/j.phrp.2020.11.2.04 PMC7104686

[13] Anon (2018) COVID-19, Australia: epidemiology report 16 (reporting week to 23:59 AEST 17 May 2020). Communicable Disease Intelligence 2020, 44. 10.33321/cdi.2020.44.45 32522141

[14] Ding Z (2020) Global COVID-19: warnings and suggestions based on experience of China. Journal of Global Health 10, 011005. 32509294 10.7189/jogh.10.011005 PMC7244931

[15] Bao C (2020) COVID-19 outbreak following a single patient exposure at an entertainment site: an epidemiological study. Transboundary Emerging Disease 0, 1–9. 10.1111/tbed.13742 32725765

[16] China NHCotPsRo (2020) COVID-19 Prevention and Control Program (Third Edition). Available at http://www.gov.cn/zhengce/zhengceku/2020-01/29/content_5472893.htm.

[17] Thompson RN (2019) Improved inference of time-varying reproduction numbers during infectious disease outbreaks. Epidemics 29, 100356. 31624039 10.1016/j.epidem.2019.100356 PMC7105007

[18] EpiEstim: Estimate Time Varying Reproduction Numbers from Epidemic Curves. R package version 2.2-1. Available at https://CRAN.R-project.org/package=EpiEstim.

[19] Rothe C (2020) Transmission of 2019-nCoV infection from an asymptomatic contact in Germany. New England Journal of Medicine 382, 970–971. 10.1056/NEJMc2001468 PMC7120970 32003551

[20] Bai Y (2020) Presumed asymptomatic carrier transmission of COVID-19. Journal of the American Medical Association 323, 1406–1407. 32083643 10.1001/jama.2020.2565 PMC7042844

[21] Nishiura H, Linton NM and Akhmetzhanov AR (2020) Serial interval of novel coronavirus (COVID-19) infections. International Journal of Infectious Disease 93, 284–286. 10.1016/j.ijid.2020.02.060 PMC7128842 32145466

[22] Hu ZL (2020) Clinical characteristics of 24 asymptomatic infections with COVID-19 screened among close contacts in Nanjing, China. Science China-Life Sciences 63, 706–711. 32146694 10.1007/s11427-020-1661-4 PMC7088568

[23] Shao P and Shan Y (2020) Beware of asymptomatic transmission: Study on 2019-nCoV prevention and control measures based on extended SEIR model. *bioRxiv* 2020.2001.2028.923169.

[24] Tang B (2020) Estimation of the transmission risk of the 2019-nCoV and its implication for public health interventions. Journal of Clinical Medicine 9, 462–475. 10.3390/jcm9020462 PMC7074281 32046137

[25] Gan H (2020) Epidemiological analysis on 1052 cases of COVID-19 in epidemic clusters. Chinese Journal of Epidemiology 41, 1004–1008. 32213270 10.3760/cma.j.cn112338-20200301-00223

[26] Chen N (2020) Epidemiological and clinical characteristics of 99 cases of 2019 novel coronavirus pneumonia in Wuhan, China: a descriptive study. Lancet 395, 507–513. 32007143 10.1016/S0140-6736(20)30211-7 PMC7135076

[27] Li Q (2020) Early transmission dynamics in Wuhan, China, of novel coronavirus-infected pneumonia. New England Journal of Medicine 382, 1199–1207. 10.1056/NEJMoa2001316 PMC7121484 31995857

[28] Anon (2020) Press Conference of the Joint Prevention and Control Mechanism of the State Council.

[29] Chen Y (2020) The epidemiological characteristics of infection in close contacts of COVID-19 in Ningbo city. Chinese Journal of Epidemiology 41, 668–672. 10.3760/cma.j.cn112338-20200304-00251 32447904

[30] Bi Q (2020) Epidemiology and Transmission of COVID-19 in Shenzhen China: Analysis of 391 cases and 1286 of their close contacts. medRxiv 2020.2003.2003.20028423. 10.1016/S1473-3099(20)30287-5 PMC7185944 32353347

[31] Mahase E (2020) China coronavirus: mild but infectious cases may make it hard to control outbreak, report warns. British Medical Journal 368, m325. 31992570 10.1136/bmj.m325

[32] Wang FS and Zhang C (2020) What to do next to control the 2019-nCoV epidemic? Lancet 395, 391–393. 32035533 10.1016/S0140-6736(20)30300-7 PMC7138017

[33] Wang DW (2020) Clinical characteristics of 138 hospitalized patients with 2019 novel coronavirus-infected pneumonia in Wuhan, China. Journal of the American Medical Association 323, 1061–1069. 32031570 10.1001/jama.2020.1585 PMC7042881

[34] Lai JW and Cheong KH (2020) Superposition of COVID-19 waves, anticipating a sustained wave, and lessons for the future. Bioessays 42, e2000178. 33040355 10.1002/bies.202000178 PMC7675615

[35] Cheong KH and Jones MC (2020) Introducing the 21st century's new four horsemen of the coronapocalypse. Bioessays 42, e2000063. 32227642 10.1002/bies.202000063

